# Discovery of tumour indicating morphological changes in benign prostate biopsies through AI

**DOI:** 10.1038/s41598-025-15105-6

**Published:** 2025-08-21

**Authors:** Eduard Chelebian, Christophe Avenel, Helena Järemo, Pernilla Andersson, Anders Bergh, Carolina Wählby

**Affiliations:** 1 Department of Information Technology and SciLifeLab, Uppsala University, 75237 Uppsala, Sweden; 2 Department of Medical Biosciences, Pathology, Umeå University, 90187 Umeå, Sweden

**Keywords:** Prostate cancer, Image processing, Machine learning

## Abstract

Diagnostic needle biopsies that miss clinically significant prostate cancer (PCa) often sample benign tissue near hidden cancers. Such benign samples might still display subtle morphological signs of cancer elsewhere in the prostate. This study examined if artificial intelligence (AI) could detect these morphological clues in benign biopsies from men with elevated prostate-specific antigen (PSA) levels to predict subsequent diagnosis of clinically significant PCa within 30 months. We analysed biopsies from 232 men initially diagnosed as benign, matched for age, diagnosis year, and PSA levels-half were later diagnosed with PCa, while the rest remained cancer-free for at least eight years. The AI model accurately predicted future PCa diagnosis from initial benign biopsies (AUC = 0.82), highlighting patterns such as changes in stromal collagen and altered glandular epithelial cells. This demonstrates that AI analysis of routine haematoxylin-eosin biopsy sections can detect subtle signs indicating clinically significant PCa before it becomes histologically apparent. Such morphological patterns shed light on the broader tissue alterations induced by prostate cancer, even in benign tissue, potentially enhancing early detection and clinical decision-making.

## Introduction

Prostate cancer (PCa) remains a significant health concern worldwide, affecting 1.4 million men annually^1^. While most cases follow a rather indolent course, some progress to aggressive tumours resulting in fatal outcomes^2^. The current diagnostic paradigm relies on raised prostate-specific antigen (PSA) in blood samples, followed by magnetic resonance imaging (MRI) and prostate needle biopsies^3^. However, due to the low specificity of PSA tests, false negative MRI and the limited coverage of needle biopsies, these may fail to detect PCa even when an aggressive tumour is present^4–7^. Consequently, there is a need for novel diagnostic and prognostic markers capable of identifying early changes associated with the presence of a clinically significant PCa. Such changes could be detected in apparently histologically normal tissue adjacent to tumours.

Multiple studies have shown that the benign parts of a tumour-bearing organ and a non-tumour-bearing organ are different^8–10^. A common explanation to this is the cancer field hypothesis which states that main parts of an organ are affected by carcinogenic agents resulting in precancerous genetic changes in the epithelial compartment^11^. Alternatively, to grow and spread, tumours need to instruct surrounding normal tissue to assist. Bergh and colleagues demonstrated alterations in the epithelial and stroma compartments of the tumour-bearing prostate in fully immune-competent rats, with the nature and magnitude of the effects being associated with tumour proximity, aggressiveness and metastatic capacity^9,12–17^. Studies in patients have confirmed similar changes and related them to patient outcome^18–22^ We have termed these changes as tumour instructed (and thus indicating) normal tissue (TINT) and proposed that they could be used as novel diagnostic and prognostic markers^9^.

Recognising that prostate needle biopsies may fail to sample tumour tissue even when cancer is present, we conducted a retrospective analysis using a dataset of patients with raised PSA and whose initial biopsies only contained benign prostate tissue. We paired patients who, within 30 months after an initial benign diagnosis, were diagnosed with PCa of varying histological grades (ISUP grades) with patients who remained cancer-free for at least eight years after their initial benign diagnosis, matching them for age and PSA levels. Deep learning techniques, specifically weakly-supervised learning^23^, allow to handle the challenge of having only patient-level information, in this case the cancer grade detected in a patient at subsequent biopsy rounds. This approach enabled the analysis and comparison of the morphological changes in hundreds of patients, a task unattainable through visual inspection alone. Our hypothesis is that it is possible to identify high-risk patients that were later diagnosed with clinically significant PCa by examining their initial benign biopsies. Additionally, we analysed the histopathological features responsible for the model performance, contributing to the understanding of the morphological changes potentially induced in TINT.

## Methods

### Study design

We collected a retrospective cohort of 232 patients from the University Hospital of Umeå (*Norrlands universitetssjukhus*) in Umeå, Sweden, spanning the years 1997 to 2016. All data were anonymized at the time of collection and remain fully anonymized, preventing identification of any individuals. The study protocol was reviewed and approved by the *Regionala etikprövningsnämnden i Umeå* (DNR 2010/366-31M). Due to the retrospective nature of the study and the use of anonymized data, the *Regionala etikprövningsnämnden i Umeå* (DNR 2010/366-31M) waived the requirement of obtaining informed consent. The study was conducted in accordance to the Declaration of Helsinki.

The study focuses on men (age 65 ± 7) with raised serum PSA (11.8 ± 7.5), an initial benign diagnosis in prostate needle biopsies (excluding cases with precancerous lesions such as PIN) and with subsequent separation in diagnostic trajectories. While some patients were finally diagnosed with PCa with varying ISUP grades at subsequent biopsy rounds within 30 months, others, despite subsequent re-biopsy rounds, remained cancer-free for a period of at least eight years. The patients were paired based on similar age, year of diagnosis and PSA level upon initial diagnosis.

As patients diagnosed with ISUP 1 are usually managed with active surveillance according^3,24^, we defined a binary endpoint that mirrors this clinical decision point. Specifically, we compared (1) patients who remained cancer-free or were subsequently diagnosed with ISUP 1 (low risk) with (2) patients who were subsequently diagnosed with clinically significant cancer, i.e. ISUP>1 (high risk). A separate intermediate-risk class was not modelled because 11 and 19 ISUP 2 and ISUP 3 cases were available, respectively, which would have provided insufficient statistical power for a three-class approach.

### Image acquisition

The patients underwent standard of care needle biopsy examinations to determine their benign state. For each patient, 10–12 ultrasound-guided transrectal needle biopsies of approximately 10 mm in length were extracted at prespecified locations (systematic biopsies). MRI-guidance was not used. We digitised the available slides using the 3D Histech Pannoramic 250 scanner (3D HISTECH Ltd., Budapest, Hungary) at a magnification of 20$\times$ and a pixel size of 0.2428 $\upmu$m/pixel.

### Deep learning workflow

In artificial intelligence, problems with image-level rather than pixel-level annotations are classified as weakly-supervised. Our study fits this paradigm since the available labels reflect whether each slide originates from a patient later diagnosed with prostate cancer (PCa) or from one who remained benign, without suspicious initial biopsy findings. Specifically, our labels are determined retrospectively based on future clinical outcomes (presence or absence of PCa), enabling automated analysis of morphological differences across slides. This approach helps identify subtle histological characteristics guiding the model’s predictions.

Our workflow employs a customized implementation of the clustering constrained attention multiple-instance learning (CLAM) algorithm^23^, a state-of-the-art method that operates under the assumption that negative slides (from patients remaining benign) contain exclusively negative evidence, whereas positive slides (patients eventually diagnosed with PCa) contain at least one region indicative of positive evidence. Our analysis focuses on identifying tumor-induced non-malignant tissue (TINT) changes within these positively labeled slides.

The initial preprocessing stage involves tissue detection and segmentation from digitized biopsies. First, the digitized slides are converted to HSV color space^25^, and Otsu’s thresholding^26^ is applied on a smoothed version of the saturation channel to distinguish tissue from background. Morphological closing and an area filter are subsequently utilized to remove small non-tissue artifacts. The identified tissue regions are then divided into non-overlapping patches of size $256 \times 256$ pixels (approximately $62 \times 62\, \mu m$) at $20\times$ magnification.

An important modification from the standard CLAM implementation is our use of a ResNet18 network trained via self-supervised learning on 57 histopathology datasets^27^ to extract meaningful feature representations from each tissue patch. This contrasts with the default approach employing a ResNet50 trained on the natural image ImageNet dataset. As a result, each $256 \times 256$ patch is represented by a 512-dimensional feature vector. After patch-level feature extraction, each slide is represented as a collection of these feature vectors.

The CLAM model is then trained using the slide-level labels with the single-branch “small” architecture for 100 epochs, applying early stopping and cross-entropy bag loss for optimization. This fully weakly-supervised approach requires no manual pixel-level annotations, thereby leveraging the intrinsic morphological patterns captured within the standard haematoxylin-eosin stained biopsy images to predict future clinically significant prostate cancer diagnoses.

The implementation of the CLAM model is publicly available at: https://github.com/mahmoodlab/CLAM. The self-supervised model utilized for feature extraction can be accessed here: https://github.com/ozanciga/self-supervised-histopathology.

### Statistical analysis

The patient cohort was partitioned into a development cohort, constituting 80% of the subjects for model optimisation, using 5-fold cross validation. To assess generalisation of the best performing model on validation, we used the remaining 20% of subjects as independent test set. We ensured that slides from the same patient did not appear in different cohorts and we balance the cohorts in terms of ISUP grade, PSA level and age distribution. The performance of the model was evaluated via the area under the receiver operating characteristic curve (AUC), assessed both at slide-level and patient-level. Patient-level predictions were obtained by considering a patient high-risk if at least one of their slides was classified as high-risk. To interpret the morphological characteristics driving the classification process, we employed uniform manifold approximation and projection for dimension reduction (UMAP)^28^ on the tile-level representations. This approach enabled the identification of tumour associated changes within the initial benign biopsies by visualising the representations in a two-dimensional space.

## Results

### Baseline characteristics

**Fig. 1:**
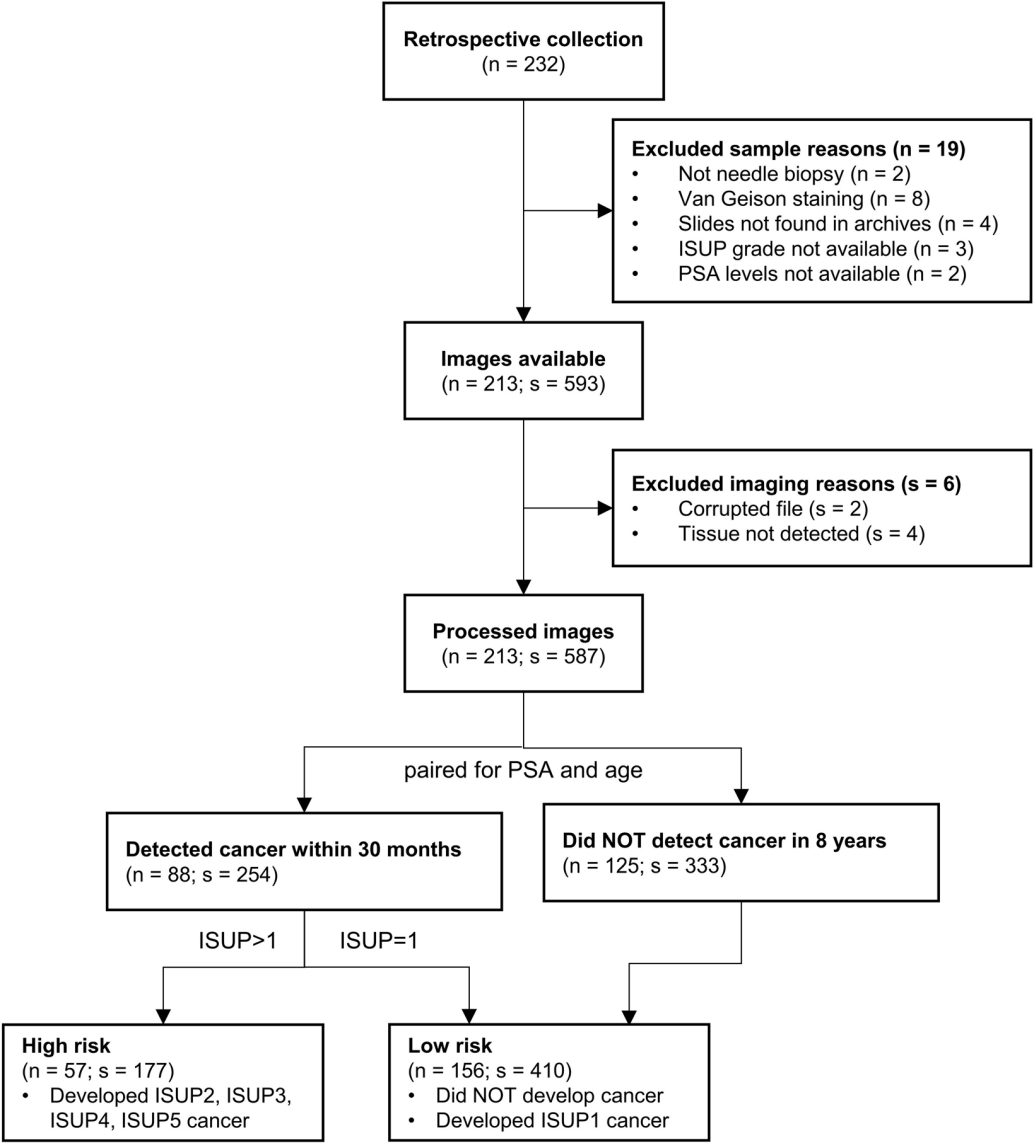
Consort diagram (n = patients; s = slides).

The final cohort included 213 patients with 587 biopsy slides as detailed in Fig. 1. 19 patients were excluded due to sample reasons, either for not being H&E-stained prostate needle biopsies or for missing data. No further patients were excluded, although six additional slides were discarded due to imaging reasons. We publicly share the final cohort in^29^.

Table 1 summarises the characteristics of the final cohort, and the partitions into the model development cohort and the test cohort. All the patients examined in this study resided in the northern region of Sweden, where the population predominantly consists of Caucasians (>90%). According to Swedish law and practice, race and ethnic origin are not recorded in patient records. Additionally, we do not have access to data regarding their co-morbidities and medications.

**Table 1:** Baseline characteristics. Percentages compared to the complete cohort per column.

	Total (n = 213)	Development (n = 170)	Test (n = 43)
Age (years)			
$<55$	15	13	2
55–60	36	30	6
60–65	55	46	9
65–70	55	40	15
70–75	33	24	9
$>75$	19	17	2
PSA (ng/mL)			
$<3$	1	1	0
3–5	21	17	4
5–10	97	76	21
$>10$	94	76	18
ISUP grade			
Benign	125	101	24
ISUP 1	31	24	7
ISUP 2	11	10	1
ISUP 3	19	14	5
ISUP 4	19	14	5
ISUP 5	8	7	1

### Model performance

**Fig. 2:**
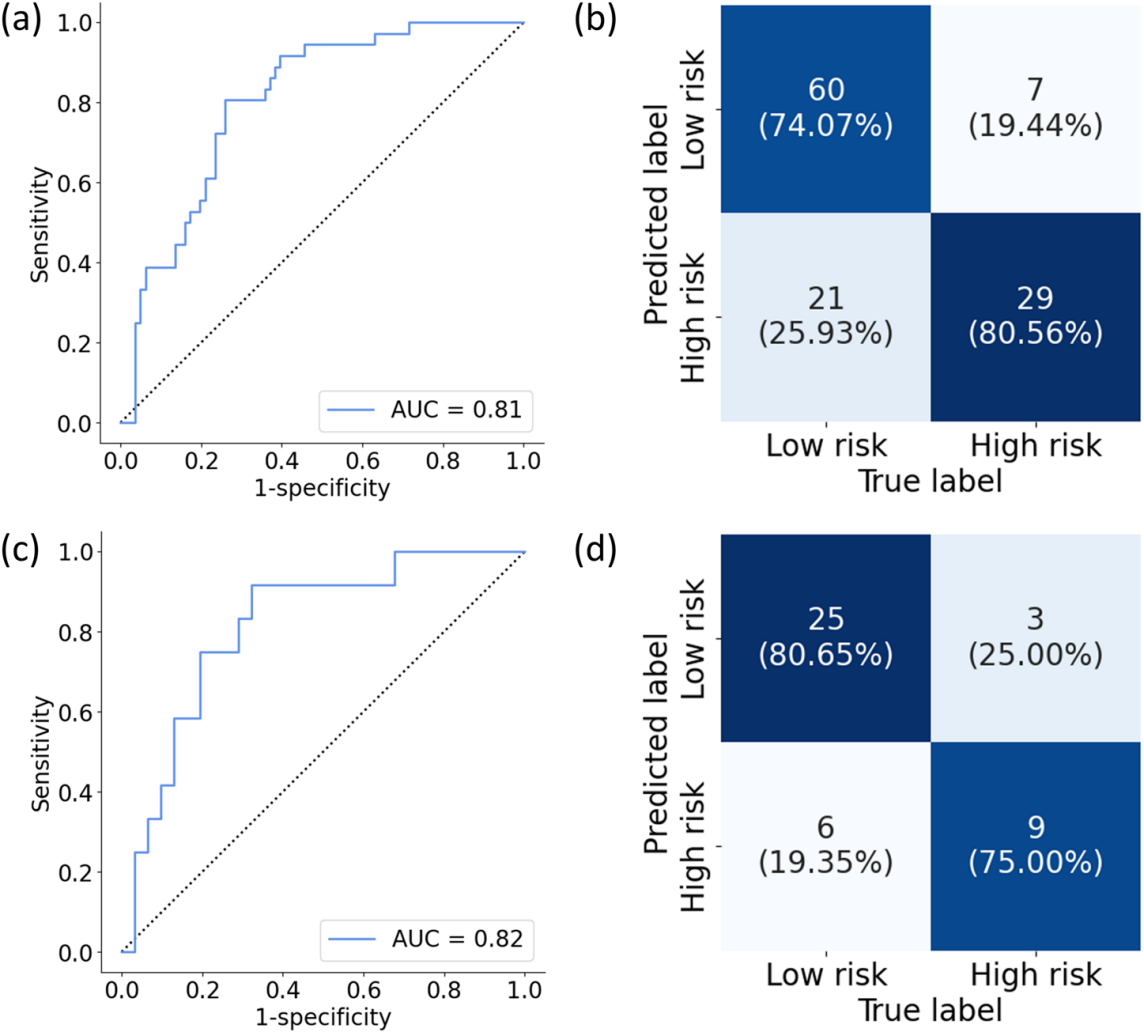
Model performance on the test set. Per slide (**a**) ROC curve and (**b**) contingency matrix. Per patient (**c**) ROC curve and (**d**) contingency matrix.

Figure 2 shows the model performance results. We identified slides coming from patients who would be diagnosed with a clinically significant PCa (ISUP2–5) with an $AUC=0.81$, and sensitivity of 0.81 at false positive rate of 0.26 on the test set. At patient level, we achieved an $AUC=0.82$ with a sensitivity of 0.92 at false positive rate of 0.32. Considering these patients were not identified at all upon initial diagnosis, we consider the sensitivity compensates for the false positive rates.

Figure 2b,d show the confusion matrices predicted patients into low-risk (cancer-free and ISUP1) and high risk (ISUP2–5). A more detailed view dividing the patients into their true ISUP class is shown in Supplementary Figure 1. Interestingly, patients who were later diagnosed ISUP1 were easier to identify as low risk than patients who continued being benign. There was also a difference of performance in identifying patients who were subsequently diagnosed with high grade PCa (ISUP4-5) and patient at intermediate risk such as ISUP 2 or ISUP 3.

### Morphological interpretation

**Fig. 3:**
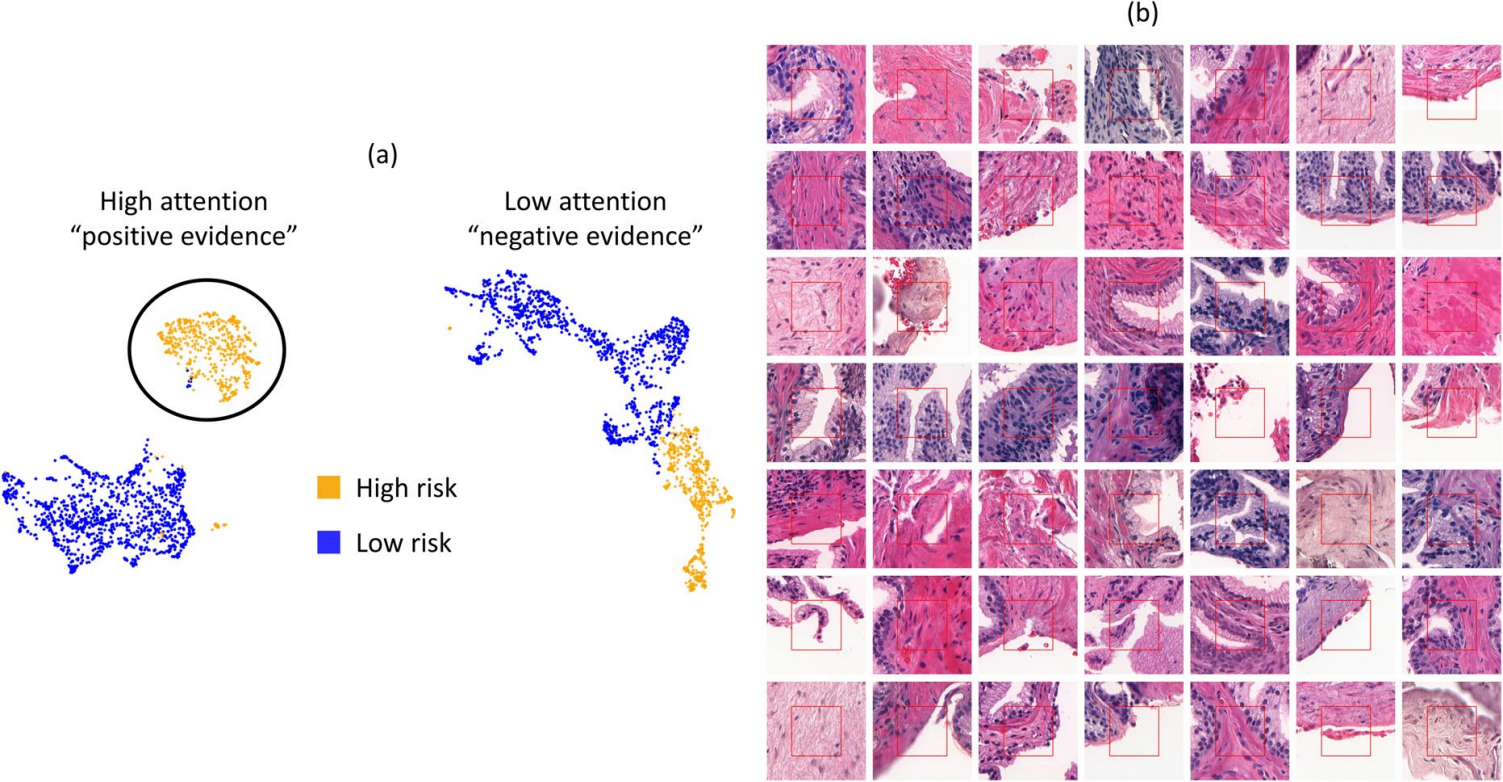
(**a**) UMAP space of the 20 most and least attended tiles from the correctly classified slides in the test set. (**b**) Sample of the most attended morphologies that drove the classification, the *positive evidence* for being high risk. The red square in the middle is the actual tile size, the rest is shown for context.

Figure 3 shows the UMAP space of the most and least attended tiles from the correctly classified slides in the test set. Tiles naturally clustered between positive and negative evidence. While low attended patches are more tightly packed regardless if they are low or high risk, highly attended tiles clearly clustered between low and high risk patients. In Supplementary Figure 2 we show the representation space for the whole test cohort.

An interactive overlay of attention regions is shared at https://prostate-attention.serve.scilifelab.se/. Note that these attention heatmaps are smoothed for better visualisation. We present the initial negative needle biopsy for two patients that were eventually diagnosed with ISUP3 and ISUP4 and the model was able to recognise them.

The sample of the morphology that drove the classification shows that changes within the tissue stroma compartment were common and informative and apparently characterised by increased collagen and reduced numbers of smooth muscle cells. While some of the top-ranked tiles did contain epithelial glands, these cues were less consistently highlighted and could be below the resolution of our down-sampled H&E images.

## Discussion

The present study suggests that AI, applied to routine H&E-stained needle biopsies, can identify morphological changes in men with elevated PSA, distinguishing between those likely to be diagnosed with significant PCa within 30 months of follow-up and those expected to remain low-risk for eight years with an AUC of 0.82. Previously, AI methods have been successfully used to detect prostate cancer in needle biopsy cores^30^ to reduce pathologist work burden by trying to replicate the diagnostic process. More recently, similar techniques have been able to differentiate tumour-adjacent benign prostate tissue in the same biopsy from prostate tissue in tumour-free men^31^. We went a step further, leveraging novel weakly-supervised learning techniques to analyse the initial benign biopsies of patients that were later diagnosed with PCa. Weakly-supervised methods have also been successful for large-scale screening in other diseases^32^.

Multiple studies have demonstrated that prostate tumours in patients are associated with changes in the benign parts of the organ^8–10^. Those changes have been detected utilising various methods, including alterations in transcriptome^10,33,34^, metabolome^35,36^, morphology^8,16–19,21,22^ or epigenetic marker expression^37,38^. Such alterations could be used to diagnose and prognosticate PCa indirectly by examining the adjacent benign parts of the prostate^9^. Specifically, they could be used to evaluate the need of re-biopsy in cases with raised PSA and initially negative prostate biopsies^9,38,39^. Additionally, previous studies in experimental models^12,13,15,40^ and in patient samples^14,17,19,21,22^ indicate that the magnitude and nature of the changes detected in the benign parts of the tumour-bearing prostate are related to tumour aggressiveness, proximity and patient outcome. For instance, the magnitude of some morphological changes, such as the increased vascular density and accumulation of macrophages adjacent to a tumour, were related to the tumour ISUP grade. Changes in benign tissue adjacent to ISUP1 tumours were more discrete while changes around ISUP 4–5 were more pronounced^17^.

Considering this background, we hypothesised that our model could differentiate changes in the benign parts of the prostate related to tumour grade, and particularly to identify high-risk cases in need of early re-biopsy. ISUP1 tumours are generally indolent so usually there is no need of early diagnosis and active treatment^24^. Importantly, our model could identify such cases and notably an ISUP1 tumour was by far the most common type detected among the cases with raised PSA later diagnosed with PCa. Some ISUP2 are candidates for active treatment while others are not and ISUP 3–5 tumours generally need early diagnosis and active therapy^3^. Our model could identify cases that were later diagnosed with ISUP 2–5 with high sensitivity, although it was less effective for ISUP4–5 cases. This could be due to many ISUP4–5 tumours showing high cell proliferation and rapid growth^17^ so they may have been too small and too far away from the biopsies to have a significant effect on the initial biopsies. Some cases that remained cancer-free were grouped among high-risk cases. As tumour-adjacent benign tissue is characterised by biological processes such as inflammation and wounding response^10,13,15,17,40^, inflammation or prostatitis could induce changes that could be misinterpreted as cancer-induced alterations by our model. It should be noted that the number of cases that were later diagnosed with high-grade tumours was rather low and further studies using larger cohorts are needed for more firm conclusions.

The biopsies in this study were guided by transrectal ultrasound and not MRI, as MRI-guided biopsies were not yet available at the time. Although MRI improves tumour detection, it is not perfect and does not always assess disease aggressiveness accurately^41^. Thus, our AI tool, which analyses standard tissue sections, remains valuable and cost-effective for identifying clinically significant tumours and early changes in tissues. Future advancements in imaging technologies, such as MR-PET^42^, may impact the tool’s relevance, but it still provides useful insights for prostate cancer diagnosis and monitoring.

Our model was able to identify areas within biopsies that were particularly informative to identify cases that were later diagnosed with PCa. The exact nature of these changes needs to be clarified using, for example, spatial transcriptomics or immunohistochemisty of selected key markers. However, already at this stage, it can be concluded that informative changes are identified in discrete parts of the stroma and the glandular epithelium. In informative stroma areas reduced fraction of smooth muscle cells and increased collagen/altered matrix were observed.

Interestingly, this resembles the development of a reactive stroma within prostate tumours^43^. In line with this, previous studies have shown an increased amount of hyaluronic acid and decreased androgen receptors in the stroma in the benign tissue surrounding prostate tumours^18,21^. Multiple studies have also demonstrated subtle morphological changes in the epithelial compartment in benign tumour adjacent tissue^8,9^, for example signs of increased EGF-receptors^20^ and decreased microseminoprotein-beta (MSMB)^16^. As could be suspected AI also identified alterations in the epithelial compartment that were associated with a later diagnosis of cancer, but additional studies are needed to characterize these alterations in more detail. The signals causing epithelial and stroma changes within tumours may extend far out into the surrounding tumour-bearing organ^9^. Importantly, as our AI achieved high sensitivity, it could be combined with tests for serum PSA variants^44^ that have high negative predictive value.

While patient-level splitting successfully prevented data leakage, several opportunities remain for strengthening the study. First, the model’s resilience to variations in scanners, staining protocols and patient demographics should be assessed on fully external cohorts drawn from other institutes to confirm generalisability and to determine whether domain-adaptation techniques are needed. Second, assembling larger, multi-centre datasets would allow (i) development of a three-class framework that explicitly isolates the intermediate-risk ISUP 2-3 group and (ii) a spatially-resolved analysis of tumour-induced changes in ostensibly benign tissue. Because the 12-core systematic biopsies analysed here were not image-tracked, we could not separate benign regions that lay adjacent to versus distant from the eventual tumour focus; such distance-stratified evaluation will require MRI-targeted biopsies or whole-mount prostatectomy material. Finally, our dataset contained only systematic cores, so we could not compare them with MRI-target cores-samples taken from imaging-defined lesions that may harbour subtle cancer-related alterations even when reported as negative. Prospectively studying these image-fused cores is a promising avenue for extending the model’s clinical value.

## Conclusion

In conclusion, AI was able to identify, from their initially benign H&E-stained needle biopsies, patients who were later diagnosed with clinically significant PCa. The discovered morphological characteristics can provide insights on the biological mechanisms of tumour indicating normal tissue in the tumour-bearing prostate. If these findings were confirmed, the methodology could be used as a complementary screening step.

## Data Availability

The complete clinical prostate biopsy dataset is openly accessible (https://doi.org/10.1038/s41597-025-04758-7)
